# MAP3K3 expression in tumor cells and tumor-infiltrating lymphocytes is correlated with favorable patient survival in lung cancer

**DOI:** 10.1038/srep11471

**Published:** 2015-06-19

**Authors:** Yanli He, Lihui Wang, Weijun Liu, Jinjie Zhong, Shengbin Bai, Zhuwen Wang, Dafydd G. Thomas, Jules Lin, Rishindra M. Reddy, Nithya Ramnath, Philip W. Carrott, William R. Lynch, Mark B. Orringer, Andrew C. Chang, David G. Beer, Guoan Chen

**Affiliations:** 1 University of Guangzhou Chinese Medicine, Guangzhou, China; 2 Guangxi Medical University, Nanning, China.; 3 The First People's Hospital of Yunnan Province, Kunming, China; 4 Xinjiang Medical University, Xinjiang, China; 5 Section of Thoracic Surgery, Department of Surgery, University of Michigan, Ann Arbor, Michigan, 48109, United States.; 6 Department of Pathology, University of Michigan, Ann Arbor, Michigan, 48109, United States.; 7 Division of Medical Oncology, Department of Internal Medicine, University of Michigan, Ann Arbor, Michigan, 48109, United States.

## Abstract

MAP3K3 is involved in both the immune response and in tumor progression. Its potential biological role *in vitro* in lung cancer cell lines and the association of mRNA/protein expression patterns with clinical outcome of primary lung tumors were investigated in this study. Silencing MAP3K3 using siRNA in lung cancer cell lines resulted in decreased cell proliferation, migration and invasion. These effects were associated with down-regulation of the JNK, p38, AKT, and GSK3β pathways as determined using phospho-protein and gene expression array analyses. However, MAP3K3 mRNA and protein overexpression in primary lung tumors correlated significantly with favorable patient survival. Gene cluster and pathway analyses of primary tumor datasets indicated that genes positively-correlated with *MAP3K3* are significantly involved in immune response rather than the cell cycle regulators observed using *in vitro* analyses. These results indicate that although MAP3K3 overexpression has an oncogenic role *in vitro,* in primary lung adenocarcinomas it correlates with an active immune response in the tumor environment that correlates with improved patient survival. MAP3K3 may potentially not only serve as diagnostic/prognostic markers for patients with lung cancer but also provide an indicator for future investigations into immunomodulatory therapies for lung cancer.

Lung cancer is the leading cause of cancer-related death in the USA, with an estimated 224,210 new cases and 159,260 deaths in 2014^1^. A 5-year survival rate of only 17% reflects lung cancer’s known heterogeneity, the complex cellular, molecular and tumor microenvironmental factors present in each individual and poor therapy options^1^,^2^. The most common subtypes of non–small cell lung cancer (NSCLC) are adenocarcinoma (ADC) and squamous cell carcinoma (SCC). Significant advances in understanding of the critical molecular alterations and immune microenvironment present in lung cancer have provided clinically relevant biomarkers that stratify patients according to their outcome^3^,^4^,^5^,^6^,^7^,^8^,^9^,^10^. Additionally these biomarkers have contributed to the development of novel therapeutic strategies by identifying new targets as well as predictive markers for specific drugs^11^,^12^,^13^,^14^,^15^,^16^. There remains however, an urgent need for the identification of novel molecular biomarkers that provide clinicians with useful information concerning patient prognosis and potential therapeutic options^17^,^18^.

The *MAP3K3* gene is located on 17q, encodes the mitogen-activated protein kinase kinase kinase3, and functions as an upstream regulator of the MAPK pathway, regulating many cellular functions including cell proliferation, differentiation, migration and apoptosis^19^,^20^. Biochemical studies indicate that MAP3K3 is able to activate multiple MAPKs *in vitro* including the ERK1/2, JNKs, p38 and ERK5 under certain conditions^21^,^22^. In addition, MAP3K3 can activate the IκB kinase (IKK)/NF-κB pathway^23^. Previously, MAP3K3 was reported to play an important role in early embryonic cardiovascular development, endothelial cell proliferation, muscle cell formation^24^,^25^ as well as T cell proliferation and survival^26^,^27^. It is also a crucial and specific regulator of the proinflammatory cytokines IL-6 and GM-CSF in macrophages^28^. Besides roles in the immune response, MAP3K3 has been reported to play a role in tumorigenesis, with increased expression being associated with and chemoresistance in breast cancer, esophageal and ovarian cancer^29^,^30^,^31^. The expression and roles of MAP3K3 in lung cancer, however, and its underlying molecular mechanisms remain unknown.

In this study, we investigated the roles of MAP3K3 in cell proliferation, migration, invasion and effects on cell cycle following MAP3K3 knockdown using siRNA in lung cancer cell lines. Potential MAP3K3 regulated proteins/genes were examined using both phospho-protein arrays and gene expression microarray analysis. We then analyzed the MAP3K3 protein/mRNA expression in primary lung cancers and its association with clinical-pathological characteristics including patient survival.

## Results

### Knockdown of MAP3K3 suppresses lung cancer cell proliferation, migration and invasion

**Figure 1:**
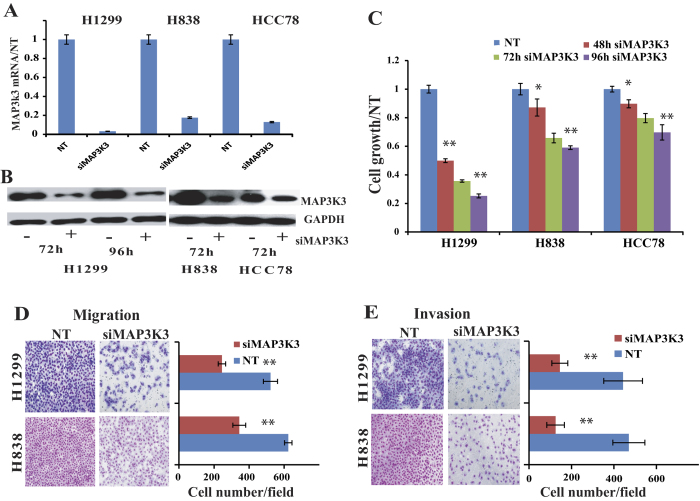
Silencing MAP3K3 decreased lung cancer cell proliferation, migration and invasion. MAP3K3 siRNA (siMAP3K3) was used to knockdown MAP3K3 expression in three lung cancer cell lines (H1299, H838, and HCC78). The non-target siRNA (NT) was used as control. The MAP3K3 expression at the mRNA (**A**) and protein (The Western blots have been run under the same experimental conditions) (**B**) levels were decreased more than 90% after MAP3K3 specific siRNA treatment compared to NT. (**C**): Knocking-down MAP3K3 significantly decreased cell growth at the time point shown compared to NT measured by WST-1. (**D**): MAP3K3 siRNA inhibited tumor cell migration, and (**E**): inhibited cell invasion in both H1299 and H838 cells at 72 h. The representative microscopy images are shown on the left (magnification: 10×). The quantification of 10 randomly selected fields is shown on the right. The values shown are expressed as the mean ± SD of three independent experiments. (*p < 0.01; **p < 0.001 versus NT).

The biological function of MAP3K3 in lung cancer is poorly understood. In order to test which lung cancer cell line is MAP3K3 dependent, we used siRNA technology to knockdown MAP3K3 expression in 9 lung cancer cell lines. We found that 5 out 9 are sensitive to MAP3K3 siRNA knockdown by proliferation (WST assay), including H1299, H838, HCC78, H1792 and H1650. The knockdown efficiency of MAP3K3 siRNA was confirmed by real-time quantitative RT-PCR (qRT-PCR) and Western blot. As compared to non-target siRNA, treatment with MAP3K3 siRNA resulted in a significant decrease in cell proliferation in these 5 lung cancer cell lines. (Three of them are shown in Fig. 1A–C). Other 4 cell lines, H1437, HCC827, H2347 and H23, are not affected by cell proliferation although the RNAs are decreased by MAP3K3 siRNA knockdown indicated that these 4 cell lines are MAP3K3 independent (data not shown). H1299 and H838 are more invasive cells using Boyden chamber assays than other cells tested therefore we selected H1299 and H838 for further experiments.

Next, we evaluated the effects of MAP3K3 knockdown on both tumor cell migration and invasion. Trans-well assays were employed using H1299 and H838 cells. Cell migration was significantly decreased with MAP3K3 siRNA as compared to the cells treated with non-target siRNA (*p* < 0.001, Fig. 1D). Consistent with the migration assay results, silencing MAP3K3 also significantly inhibited cell invasion through matrigel-coated membranes (*p* < 0.001, Fig. 1E). These results suggested that MAP3K3 is involved in lung cancer growth, migration and invasion.

### MAP3K3 knockdown affects AKT and GSK-3β signaling pathways

**Figure 2:**
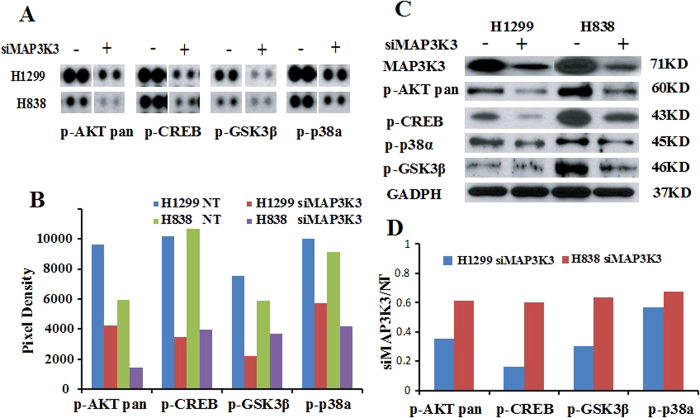
Silencing MAP3K3 down-regulates multiple proteins. (**A**): Four phospho-proteins (p-AKTpan, p-GSK3β, p-P38α and p-CREB) were down-regulated after MAP3K3 knockdown at 72 h in both the H1299 and H838 cell lines as compared to non-target siRNA control as determined using the Human MAPK antibody array (The original image is in Supplementary Figure S7), and (**B**): Quantitation of pixel densities were evaluated using Image J software. (**C**): The four proteins were then verified by Western blotting (The Western blots have been run under the same experimental conditions), and (**D**): proteins were decreased ~50% as compared to NT when analyzed by Image J software.

To investigate possible cellular pathways affected by MAP3K3 silencing, we used Human Phospho-Mitogen-activated Protein Kinase (MAPK) antibody arrays to detect potential altered expression of 26 protein kinases using both H1299 and H838 lung cells. As shown in Fig. 2A,B, following treatment with MAP3K3 siRNA, expression of phospho-AKTpan, GSK-3β, P38α and CREB was down-regulated as compared to non-target control. These results were then confirmed using Western blot analysis and as shown in Fig. 2C,D, phospho-AKTpan, GSK-3β, P38α and CREB protein expression were reduced after MAP3K3 knockdown, consistent with the results of the protein kinase antibody arrays. This indicated that MAP3K3 knockdown suppressing tumor cell growth and invasion may through regulate AKT and GSK-3β pathways.

### MAP3K3 regulates genes affecting G1 cell cycle arrest via CDC25A and CCNE1 regulation

**Figure 3:**
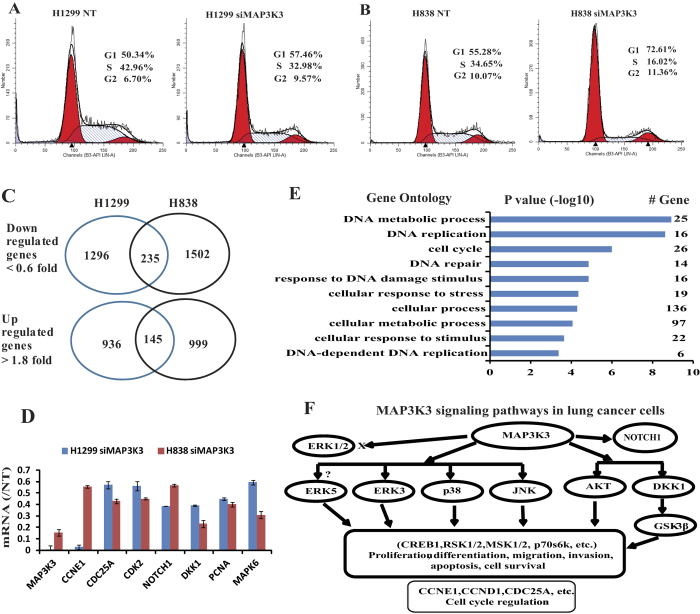
MAP3K3 regulates multiple pathways/genes affecting cell cycle at G_1_ arrest and potentially through CDC25A and CCNE1 regulation in lung cancer cells. Knockdown using MAP3K3 siRNA caused G_1_ arrest in both (**A**) H1299 and (**B**) H838 lung cells. (**C**): MAP3K3 regulates many genes in both H1299 and H838 cells, with verification by qRT-PCR (**D**). (**E**): DAVID pathway analysis of 235 (206 found in DAVID) down-regulated genes in Fig. 3C showing that these genes are significantly associated with: DNA metabolic, DNA replication and cell cycle processes (*p* > 6 of –log10). (**F**): MAP3K3 potentially regulated pathway/genes in lung cancer cells as determined by protein phospho-array and gene expression array analyses in this study. ERK1/2 and ERK5 protein/mRNA were not changed after MAP3K3 siRNA treatment.

To help determine how MAP3K3 affects tumor growth, we utilized flow cytometry analysis to examine the effect of MAP3K3 knockdown on the cell cycle. As shown in Fig. 3A,B, knockdown of MAP3K3 using siRNA induced cell cycle arrest at the G1 phase indicating that G1 arrest might be one mechanism for how MAP3K3 knockdown decreases lung tumor cell growth.

To investigate potential genes specifically altered after MAP3K3 siRNA knockdown, we performed Affymetrix ST2.1 exon arrays to detect the changes in the whole transcriptome. As shown in Fig. 3C, 1296 and 1502 genes were down-regulated by at least 0.6 fold (relative to non-target siRNA control) in H1299 and H838 cells, respectively, and 235 genes were down-regulated in both cell lines including some important cell growth genes such as *PCNA, DKK1, NOTCH1, ERK3, MAPK6, CDK2, CCND1/2, CDC25A* and *CCNE1*. Several were validated using qRT-PCR (Fig. 3D). Cell cycle related genes (Supplementary Figure 1) were decreased after MAP3K3 knockdown supporting the premise that MAP3K3 may affect the cell cycle at G1/S through regulation of *CDK2, CDC25A, CCND1/2* and *CCNE1* genes. Further verification of these genes is warranted.

Potential pathways were examined using DAVID analysis^32^, and the Gene Ontology of the top 235 (206 found in DAVID) down-regulated genes indicated that these genes are significantly involved in: DNA metabolic process, DNA replication and cell cycle (*p* > 6 of −log10) (Fig. 3E). There were also 936 and 999 genes up-regulated at least 1.8-fold in H1299 and H838 cells, respectively, with 145 genes up-regulated in both cells. We found 14 microRNAs, including miR222 and miR421, up-regulated upon MAP3K3 knockdown. DAVID pathway analysis however did not indicate a pathway or Gene Ontology that was significantly involved.

Taken together, these results suggest that *in vitro* MAP3K3 influences lung tumor growth, migration and invasion through multiple pathways. These include the previously documented association with the JNK, p38, AKT and GSK pathways, and from our results, potentially also the MAPK6 (ERK3) and NOTCH1 pathways (Fig. 3F). MAP3K3 in lung cancer cells is not involved in the ERK1/2 pathway as reported in T cell or breast cancer^26^,^29^ thus suggesting potential tissue specificity. We also did not find that MAPK7 (ERK5) protein/mRNA was changed after MAP3K3 knockdown. However, we suggest that MAP3K3 may regulate the cell cycle through effects on *CDC25A*, *CCND1/2* and *CCNE1* gene regulation (Fig. 3F).

### MAP3K3 protein expression is present in both primary lung adenocarcinomas and in tumor-infiltrating lymphocytes

**Figure 4:**
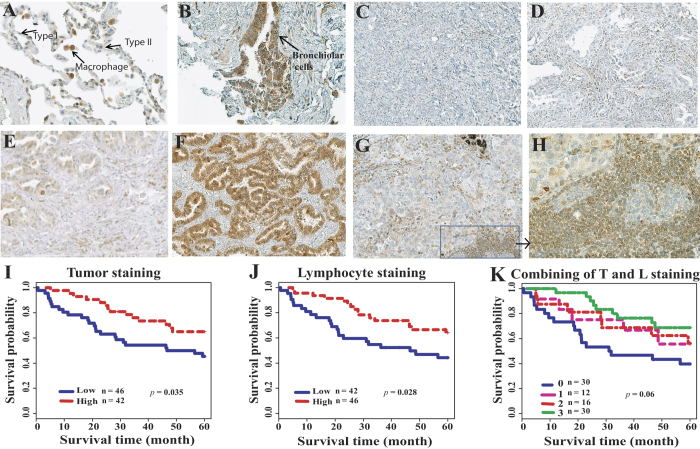
Representative immunohistochemical staining of MAP3K3 protein in lung tissues and correlation with patient survival. Immunostaining of MAP3K3 in normal lung tissue (**A**) and (**B**), lung ADC tissues (**C**)–(**F**), and tumor-infiltrating lymphocytes (**G**) and (**H**). Original magnifications: (**A**), (**B**) and (**H**) x40, others are x20. (**I**): MAP3K3 positive tumor staining (score 2 and 3) is favorable for patient survival as compared to negative or weak staining (score 0 and 1) as determined by log-rank test. (**J**): MAP3K3 positive tumor-infiltrating lymphocyte staining (score 2 and 3) is favorable for patient survival as compared to negative or weak staining. (K): Both ADC (T) and lymphocyte (L) positive staining ADCs (#3 inside the figure) have better survival as compared to other groups in generally (*p* = 0.06), and *p* = 0.01 when both stained positive (#3 inside the figure, n = 30/88, 34.1%) versus both negative (#0 inside the figure, n = 30/88, 34.1%). Patient survival with staining T positive and L negative (#1 inside the figure, n = 12/88, 13.6%) or T negative and L positive (#2 inside the figure, n = 16/88, 18.2%) is in the middle.

To examine MAP3K3 protein expression in primary lung cancers, immunohistochemistry analysis with a specific anti-MAP3K3 antibody was performed. Tissue microarray arrays (TMA) containing 88 cases of lung adenocarcinoma tissues and 15 cases of normal lung tissue were examined. The MAP3K3 protein was detected mainly within the cytoplasm of primary tumors, with a few samples demonstrating positive nuclear staining (Fig. 4). In normal lung tissues, type 1 alveolar cells showed negative or very weak MAP3K3 protein staining, whereas bronchiolar cells, macrophages, fibroblasts and alveolar type 2 cells express MAP3K3 (Fig. 4A,B). Among the 88 tumors, protein scores of 14 (15.9%), 33 (37.5%), 30 (34.1%) and 11 (12.5%), representing 0 (negative), 1 (weak), 2 (moderate) and 3 (strong) expression of MAP3K3, respectively, were observed (Fig. 4C–F).

Interestingly, we found that approximately half of the tumors (43/88, 48.9%) had infiltration of lymphocytes cells within the tumor, and 100% of these 43 cases demonstrated very positive staining of these lymphocytes (Fig. 4G,H). This supports the premise of MAP3K3 involvement in T cell proliferation and survival^26^,^33^ and the notion that increased T cells both within and surrounding the tumors may increase antitumor immunity^34^,^35^. Further we observed that among the 43 MAP3K3 lymphocyte-positive (score 2 and 3) samples, 27 (62.8%) tumors also demonstrated positive staining (score 2 and 3) in tumor cells. This was significantly higher than the MAP3K3 tumor staining in the negative (score 0 and 1) lymphocyte group (14/45, 31.1%, *p* = 0.0006) (Supplementary Table S1).

The correlation between MAP3K3 protein expression (score 0 and 1 vs. score 2 and 3) and specific clinical-pathological variables is summarized in Supplementary Table S1. Positive (scores 2 and 3) of MAP3K3 protein expression was higher in well-differentiated tumors (17/26, 65.4%) as compared to poorly differentiated tumors (10/29, 34.5%, *p* = 0.02). MAP3K3 positive tumors with scores of 2 and 3 (42/88, 47.7%) showed a longer survival trend (*p* = 0.035) as compared to tumors with scores of 0 and 1 (46/88, 52.3%) (Fig. 4I). Interestingly, the 43 tumors having lymphocyte positive staining also showed a longer survival trend (*p* = 0.028) (Fig. 4J). The favorable survival trend was clearer even for positive staining of MAP3K3 in both tumor and lymphocytes versus MAP3K3 negative staining in both samples (Fig. 4K, *p* = 0.01 for both positive vs. both negative). Taken together, MAP3K3 protein overexpression is associated with favorable patient survival, potentially reflecting greater lymphocyte infiltration into tumors. Further analysis of MAP3K3 protein expression with a large series of primary lung tumors is warranted.

### *MAP3K3* mRNA overexpression correlates with favorable survival in patients with lung adenocarcinomas

**Figure 5:**
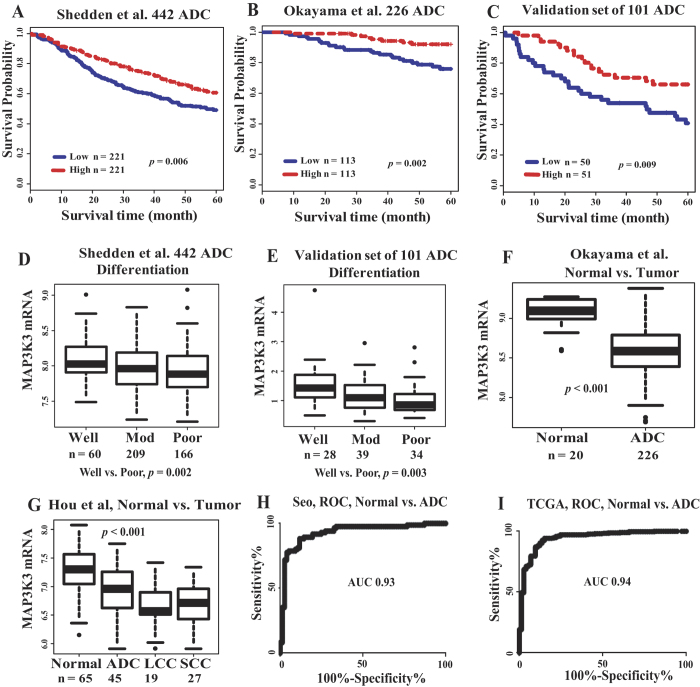
*MAP3K3* mRNA expression in ADC. Kaplan-Meier survival curves showing MAP3K3 mRNA overexpression correlates with favorable survival for patients with ADC in Shedden *et al.*, 442 ADC dataset^5^ (**A**), Okayama *et al.*, 226 ADC dataset^36^ (**B**), and verification by qRT-PCR in an independent set of 101 ADC (**C**). Median value of MAP3K3 mRNA was used as the cutoff to separate patients into high or low groups and examined using log rank. (**D**): MAP3K3 mRNA is higher in well-differentiated tumors in the Shedden *et al*., dataset and verified by qRT-PCR in an independent set of ADC (**E**). (**F**): MAP3K3 mRNA is higher in normal lung tissues in the Okayama and Hou *et al.*, (**G**) datasets^37^. (**H**): The AUC from ROC curves (normal vs. ADC) is 0.93 in the Seo *et al*., RNA-seq dataset^38^, and 0.94 in the TCGA RNA-seq dataset (**I**)^3^.

*MAP3K3* mRNA expression and its relationship to clinical-pathological variables, including patient survival in lung cancer tissues, has not been reported. We first performed analysis of *MAP3K3* mRNA expression and patient survival in two of the largest published lung microarray data sets containing 668 primary lung ADC (Supplementary Table S4): Shedden *et al.*, including 442 stage 1 to 3 ADCs^5^, and Okayama *et al.*, including 226 stage 1 and 2 ADCs^36^. Consistent with MAP3K3 protein expression, Kaplan-Meier survival curve analysis with the log-rank test indicated that patients with increased *MAP3K3* mRNA expression had a significantly longer survival probability, whereas those with low *MAP3K3* expression had a poorer survival (Fig. 5A,B) in both data sets (*p* = 0.006 and 0.002, respectively). Furthermore, multivariate Cox proportional hazards model analysis revealed that *MAP3K3* mRNA remained a significant independent favorable prognostic factor after adjusting for age, gender, stage and tumor differentiation status (*p* = 0.03 and 0.003, respectively) (Supplementary Table S2). To verify these microarray-based findings, we performed qRT-PCR analysis of *MAP3K3* expression on an independent set of 101 ADCs, including stages 1 to 3. Consistent with the two published microarray datasets, increased *MAP3K3* mRNA was significantly related to a favorable survival in this validation ADC cohort (Fig. 5C, *p* = 0.009) (Supplementary Table S2).

We then analyzed *MAP3K3* mRNA expression and other clinical variables using the Shedden *et al.*, 442 ADC cohort, since this dataset contained additional clinical information not available in the Okayama dataset. *MAP3K3* mRNA level was significantly higher in well-differentiated tumors as compared to poorly-differentiated tumors (Fig. 5D, *p* = 0.002). This was also confirmed in our qRT-PCR of 101 ADCs (Fig. 5E, *p* = 0.003).

Interestingly, we found that *MAP3K3* mRNA was higher in normal lung tissues compared to lung cancer in two published microarray data sets^36^,^37^ (Fig. 5F,G) and two RNA-seq data sets^3^,^38^ totaling 923 lung tumors and 235 normal lung tissues (Supplementary Figure S2). The AUC (area under the curve) values (normal vs. ADC) from ROC (receiver operating characteristic) curve analysis are 0.93 and 0.94, respectively, for these two RNA-seq datasets (Fig. 5H,I) indicating that MAP3K3 may potentially be used as diagnostic marker in lung cancer. Using the Oncomine database, we observed that *MAP3K3* mRNA was decreased in many other cancers (vs. normal) including bladder, breast, esophagus, head and neck, liver and prostate cancers, whereas, colorectal cancer, leukemia, lymphoma, myeloma and ovarian cancers were not consistent from different studies (Supplementary Figure S3).

In addition, we investigated *MAP3K3* DNA copy number changes, mutation, gene fusion and their relationship in lung cancer. *MAP3K3* DNA was found to be amplified in 20–30% of breast cancers^29^, but we only found 4 tumors with single copy gains and 1 tumor with heterozygous deletion among 90 ADCs analyzed by SNP6.0 Affymetrix SNP arrays (Supplementary Figure S4, unpublished data). *MAP3K3* gene mutations were detected in 16 out of 1218 (1.3%) lung cancers from the Sanger Sequencing Center and in our data (Supplementary Figure S5). The *MAP3K3* promoter was not hypermethylated in lung cancer^8^. Interestingly, we found one *MRC2-MAP3K3* fusion gene in the H1734 lung cancer cell line among 100 RNA-sequenced lung tumors and verified by RT-PCR^4^. The functional characterization of this fusion gene is warranted. By literature review, there are 6 cases of *MAP3K3* gene fusions in lung, breast and other diseases^4^,^29^,^38^,^39^,^40^,^41^ (Supplementary Table S3). *MAP3K3* DNA changes are present in a small subset of lung cancers but are not associated with outlier mRNA expression (data not shown).

Taken together, these results clearly show that overexpression of *MAP3K3* mRNA is significantly higher in normal lung tissues and in well-differentiated tumors and is associated with favorable survival in patients with lung ADC. MAP3K3 overexpression may serve as a potential biomarker for diagnosis and prognosis of ADC patients in the clinical setting.

### MAP3K3 expression correlates with PRC2, TGF-β and EMT down-regulated signatures and regulates the immune response in primary lung cancer

The favorable relationship of MAP3K3 expression in our primary tumor analysis and patient survival appears in contrast with *in vitro* results demonstrating that MAP3K3 promotes tumor growth, migration, invasion and cell cycle regulation in lung cancer cell lines and has an oncogenic role in other cancer cells^29^,^30^,^31^. To further understand the potential basis for this difference we performed Spearman correlation analysis of *MAP3K3* mRNA with whole transcriptome genes based on the Shedden *et al.*, 442 ADCs. There were 504 positively and 590 negatively correlated genes (Spearman ρ > 0.3 or < −0.3, n *=* 442, *p* < 0.0001, FDR = 0.01).

**Figure 6:**
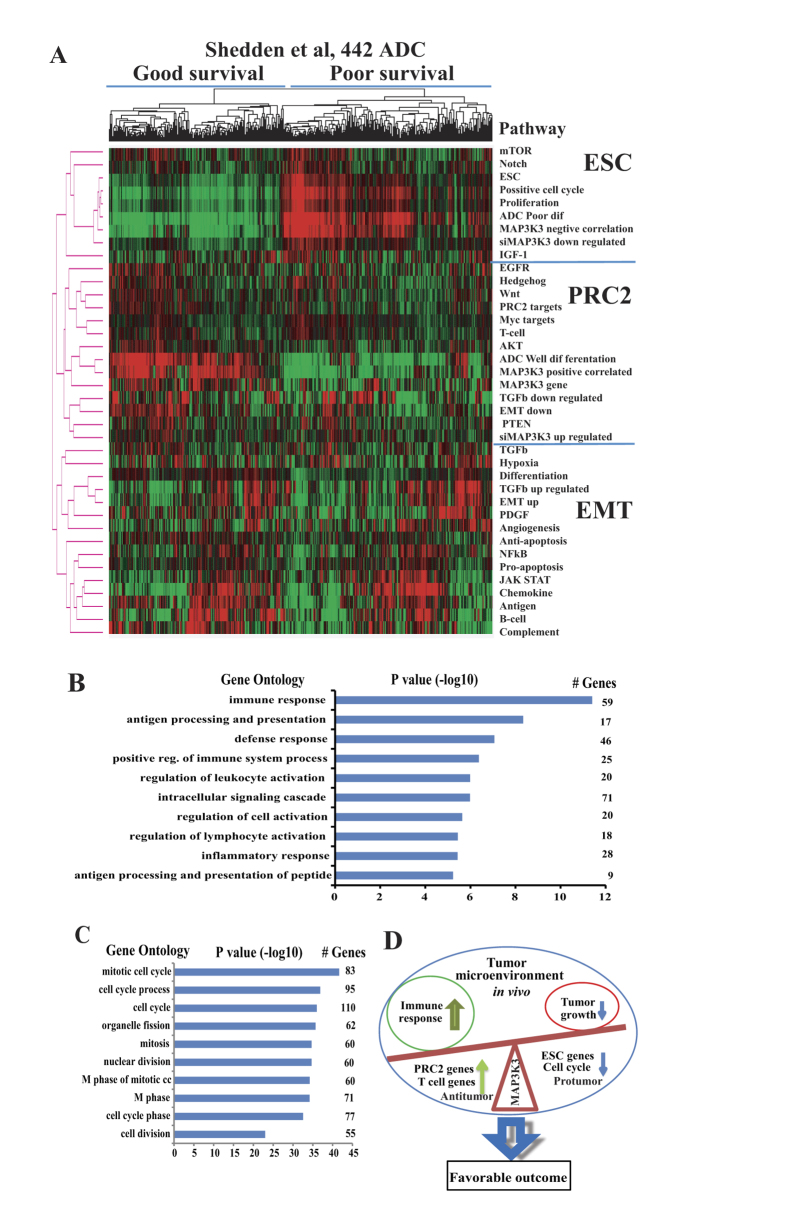
Cluster and DAVID pathway analysis of MAP3K3 mRNA correlated genes. (**A**): Heat maps showing the unsupervised cluster analysis of MAP3K3 correlated (positive or negatively, based on Shedden *et al.*, 442 ADCs by Spearman correlation) genes, MAP3K3 regulated (after MAP3K3 siRNA knockdown of H1299 and H838 cells) genes, most cancer related pathways, ESC (embryonic stem cell), PRC2 (polycomb repressive complex 2) and EMT (epithelial-mesenchymal transition) related gene signatures (mean expression value of each pathway or gene group was used). Three major clusters were found, ESC, PRC2 and EMT, based on their mRNA expression patterns. X-axis represents samples, y-axis pathways/*MAP3K3* gene, red represents high expression and green low expression, (**B**): DAVID pathway or Go Ontology analysis of these *MAP3K3* correlated genes indicates that the *MAP3K3* positive correlated genes were significantly involved in immune response (*p* > 10 of –log10 value), and (**C**): *MAP3K3* negatively correlated genes were significantly involved in cell cycle related process (*p* > 30 of –log10 value. (**D**): Potential working model for MAP3K3 in the tumor microenvironment of lung cancer. MAP3K3 overexpression may be beneficial for patient survival due to the change of balance between active immune response and tumor growth in the tumor environment.

We first performed an unsupervised cluster analysis of these *MAP3K3* correlated (positive or negative correlated) genes together with MAP3K3 regulated genes (after MAP3K3 siRNA knockdown in H1299 and H838 cells), many cancer related pathways, ESC (embryonic stem cell), PRC2 (polycomb repressive complex 2), TGF-β regulated and EMT (epithelial-mesenchymal transition) related signatures^42^,^43^. As shown in Fig. 6A, MAP3K3 negatively correlated genes (*in vivo*) and down-regulated genes (*in vitro*, after MAP3K3 siRNA knockdown) were clustered with ESC, positive cell cycle and proliferation signatures, (ESC cluster). Whereas, MAP3K3 positively correlated genes and up-regulated genes were clustered with EMT down-regulated, PRC2 targets, T cell related, TGF-β down-regulated and genes increased in expression in well-differentiated tumors, (PRC2 cluster). This suggested that MAP3K3 positively regulated genes *in vitro* (such as positive cell cycle in Fig. 3E) are either reduced or become negatively correlated to *MAP3K3 in vivo*. These included some important genes such as *MAPK6* (*ERK3*), *PCNA*, *BIRC5* and *TRIP13* (Supplementary Figure S6).

Further, we performed DAVID pathway or GO Ontology analysis on these *MAP3K3* positively or negatively correlated genes. Surprisingly, we found that the *MAP3K3* positively correlated genes in primary tumors were significantly involved in the immune response (Fig. 6B, *p* value (−log10) = 11 to 14 for top 3 groups). Survival analysis of these 59 immune response genes, we found that all these 59 genes have a favorable survival trend with 46/59 having *p* < 0.05 in Shedden *et al*., 442 lung ADCs (data not shown). The typical genes such as *CD4* and *CX3CL1* were reported to related to favorable survival^10^ or inhibit tumor growth *in vivo*^44^. This indicates that MAP3K3 is involved in the immune response in primary lung cancer, which is also supported by recent studies in T cells^22^,^26^,^28^. Whereas, *MAP3K3* negatively correlated genes *in vivo* were significantly involved in cell cycle related processes (Fig. 6C, *p* value (−log10) = 25 to 35 for the top 3 groups) such as *CCNA2*, *CCNB1/2*, *CDK1*, *CDC20* and *CDC25A*, etc (data not shown). This suggests that the gene signatures regulated by MAP3K3 differ *in vitro* and *in vivo* due to the altered tumor microenvironment the overexpression of MAP3K3 correlating with a favorable patient outcome reflects an active immune response in which related anti-tumor genes kill more tumor cells and hence improve patient survival.

## Discussion

The *MAP3K3* gene is amplified in 8–20% of breast cancers, and knockdown of its expression inhibits cell proliferation and colony formation in *MAP3K3*-amplified breast cancer cell lines^29^. Knockdown of MAP3K3 expression sensitized *MAP3K3*-amplified breast cancer cells to apoptosis induced by TNFα, TRAIL, doxorubicin, VP-16 and fluorouracil^29^. We did not find *MAP3K3* gene amplification in lung cancer, but consistent with breast cancer, silencing MAP3K3 significantly reduced lung cancer cell proliferation, migration and invasion. MAP3K3 contributes to lung cancer aggressive behavior *in vitro* potentially through regulation of multiple pathways including the JNK, p38, and AKT pathways as reported by others^23^,^26^,^29^,^31^, and as suggested by our results the DKK1-WNT-GSK, ERK3 and NOTCH1 pathways (Fig. 3F). Importantly, in lung cancer MAP3K3 is not involved in the ERK1/2 pathway which was reported in T cell or breast cancer^26^,^29^ or the MAPK7 (ERK5)^45^ pathway, potentially reflecting tissue specificity. Furthermore, our study revealed that MAP3K3 regulates the cell cycle through effects on CDC25A, CCND1/2 and CCNE1 regulation in lung cancer. The MAP3K3-related pathways discovered by phospho-protein and gene expression arrays in lung cancer cells warrant further study.

The MAP3K3 protein was detected in 63% (17/27) of primary ovarian cancers^31^, and 4/6 of primary breast cancers^29^. The MAP3K3 protein is expressed in 34/61 (55.7) and 67.7% (63/93) of samples of esophageal dysplasia and cancer, respectively and its overexpression is related to reduced median disease-free survival^30^. However, these two studies didn’t report if MAP3K3 is expressed in infiltrated lymphocytic cells. In this study, MAP3K3 protein expression was detected in 47.7% (42/88) of lung ADC with an IHC score of 2 and 3 and was significantly higher in well-differentiated tumors and in tumors demonstrating lymphocytic cell infiltration. Surprisingly, we found that the MAP3K3 positive tumors were associated with increased patient survival, which is opposite the case with esophageal cancer^30^. This could be partially due to different tumor microenvironment such as more lymphocytes infiltrating into the tumor environment in lung cancer, or reflect tissue specificity. Consistent with MAP3K3 protein expression, overexpression of *MAP3K3* mRNA was also significantly higher in normal lung tissues and well-differentiated tumors and was associated with favorable survival in patients with lung ADC in several data sets that total more than 1000 lung cancer samples and also in our RT-PCR verification analysis.

Su and others^22^,^26^,^28^,^46^ reported that MAP kinases are involved in all aspects of the immune response, from the initiation phase of innate immunity to activation of adaptive immunity. MAP3K3 is required for T cell proliferation and survival and negatively regulates the TGF-β pathway in T cells^26^,^33^. In this study, MAP3K3 promoted tumor growth, migration, invasion and cell cycle regulation in lung cancer cell lines *in vitro*, but MAP3K3 overexpression in primary lung tumors *in vivo* was correlated with increased patient survival. We hypothesize that the effects of MAP3K3 on the immune response, and tumor growth in the localized environment of the primary tumor may have a major effect on the patient’s final outcome^9^,^10^. In our cluster analysis of MAP3K3-correlated genes with ESC, PRC2, EMT, TGF-β and other cancer-related pathways, we found that *MAP3K3* positively correlated genes have the same expression pattern as PRC2 targets, T cell-related, TGF-β down regulated, increased differentiation and MAP3K3 up-regulated genes (*in vitro*, after MAP3K3 siRNA treatment) (Fig. 6A). Furthermore, we found that these positively correlated genes were significantly involved in the immune response (Fig. 6B), such as *CD4* and *CX3CL1*, etc. This indicates that overexpression of MAP3K3 correlating with a favorable patient outcome, perhaps by promoting an active immune response to anti-tumor growth that leads to improved patient survival. Further analysis of the types of tumor-infiltrating immune cells and which genes are regulated by MAP3K3 in both tumor and immune cells in the tumor microenvironment *in vivo* are warranted.

In this study, we have comprehensively investigated the role of MAP3K3 in lung cancer both *in vitro* and in the primary tumor *in vivo.* MAP3K3 is required for both tumor cell growth and for lymphocyte or other immune cell activity via several pathways. MAP3K3 overexpression correlates with a favorable patient outcome, and this may be due to the balance between an active immune response and tumor cell growth in the tumor microenvironment (Fig. 6D). Higher MAP3K3 expression in normal lung tissues and well-differentiated tumors indicates its potential use as a diagnostic as well as a prognostic marker in lung cancer. Further investigation of the potential basis for the differential influence of this gene *in vitro* from its effects *in vivo* will examined in our future studies as well as how this pathway can be utilized therapeutically for improving lung cancer patient survival.

## Methods

### Patients and samples

The lung cancer and paired non-tumoral lung tissues were obtained from patients undergoing curative cancer surgery during the period from 1991 to 2012 at the University of Michigan Health System. None of the patients included in this study received any preoperative radiation or chemotherapy. All the patients provided informed consent, and all experimental protocols were approved by the University of Michigan Institutional Review Board and Ethics Committee. The methods were carried out in accordance with approved guidelines. Resection specimens were frozen in liquid nitrogen and then stored at −80 °C until use. Other portions of the tissues were fixed in 10% formalin and embedded in paraffin for histopathological analysis. Frozen tissues for regions containing a minimum of 70% tumor cellularity were utilized for DNA/RNA/protein isolation. The median follow-up time was 8.12 years among the patients that remained alive.

### RNA isolation and quantitative RT-PCR

Total RNA was isolated from tissue samples and cell lines followed by column purification using the miRNeasy Mini kit (Qiagen) according to the manufacturers’ instructions. cDNA was prepared from RNA samples using the High Capacity cDNA Reverse Transcription kit (Applied Biosytems) according to the manufacturer’s instructions. Quantitative real-time reverse transcription-polymerase chain reaction (qRT-PCR) was prepared using Power SYBR Green master Mix (Life Technology Inc.), and qRT-PCR was performed with an ABI StepOne Real-Time PCR System (Applied Biosystems). Each sample had a final volume of 15 μL containing approximately 20 ng of cDNA. The oligonucleotide primers for *MAP3K3* were as follows: 5’-AAGGGGTCAAAGGTGGAACC-3’ (forward) and 5’-TGCCTTGAT GACGCCGTATT-3’ (reverse). Beta actin expression was used to standardize the *MAP3K3* results. Relative mRNA levels were assessed using the 2 delta Ct method^47^.

### Published microarray and RNA sequencing data collection

Three published Affymetrix microarray data sets representing 759 primary lung cancers and 65 normal lung tissues were utilized. These included Shedden *et al.* 442 stage 1 to 3 ADCs^5^, Okayama *et al.*, 226 stage 1 and 2 ADCs^36^, and Hou *et al*., 91 NSCLC and 65 normal lung tissues^37^. The basic clinical information is provided in Supplementary Table S4. The CEL files of microarray data were normalized using the Robust Multi-array Average (RMA) method^48^. We also obtained two RNA sequencing (RNA-seq) data sets^3^,^38^ consisting of a total of 923 lung tumor and 235 normal lung tissues (Supplementary Table S5). Expression levels of transcripts were represented as reads per kilobase per million mapped reads (RPKM)^49^. Our primary outcome was overall survival, censored at 5 years. The information concerning adjuvant chemotherapy or radiation therapy was provided in the original papers.

### Immunohistochemistry analysis of MAP3K3 in ADC tissue microarray

Sections of tissue microarrays (TMA) constructed using formalin-fixed, paraffin-embedded tissues from 88 ADC patients were cut at a thickness of 5 microns. Immunohistochemical staining was performed on the DAKO auto-stainer using DAKO LSAB+. After being deparaffinized and rehydrated, antigen retrieval was performed using preheated 10 mmol/L citrate buffer (pH 6) treatment for 10 min at 95 °C. The TMAs were incubated with the MAP3K3 primary antibody (at a 1:200 dilution, Abcam). The sections were visualized with 3,3′-diaminobenzidine and lightly counterstained with hematoxylin. Evaluation of the immunohistochemistry was scored independently by two pathologists using a semi-quantitative method based on staining intensity with a score of 0 (negative staining), 1 (weak staining), 2 (moderate staining) and 3 (strong staining). Based on the MAP3K3 expression levels, the ADC patients were divided into two groups: a negative or low MAP3K3 expression group (score 0 or 1) and a positive or high MAP3K3 expression group (score 2 or 3).

### Protein extraction and Western blot analysis

Protein extraction was performed as previously described. Briefly, both the ADC samples and the cell lines were homogenized in a RIPA lysis buffer, and the lysates were cleared according to previous methods. After blocking for 1 h with 5% non-fat milk, the membranes were incubated with primary monoclonal antibodies against MAP3K3 (0.2 μg/ml), Human Phospho-AKT (1 μg/ml), GSK-3β (1 μg/ml), p38α (1 ug/ml), Human Phospho-CREB Affinity Purified PAb (0.5 μg/ml), or GAPDH (at a 1:10000 dilution) overnight at 4 °C. After incubation with HRP-conjugated secondary antibody (at a 1:2000 dilution) for 1 h at room temperature, the membranes were developed using ECL and exposed to X-ray film.

### Cell culture and siRNA transfection

Human lung cancer cell lines were obtained from American Type Culture Collection (ATCC) and cultured in RPMI1640 medium supplemented with 10% FBS at 37 °C in a humid atmosphere consisting of 5% CO2/ 95% air. To knockdown MAP3K3, these cells were treated with 10 nM ON-TARGET plus SMART pool Human MAP3K3 siRNA or non-target control siRNA #1 and #5 (Thermo Scientific Dharmacon). Transfections were performed using the Lipofectamine RNAi MAX reagent (Invitrogen, USA) according to the manufacturer’s instructions.

### Cell proliferation assay

The WST-1 cell proliferation Assay (Roche) was used to measure cell proliferation after MAP3K3, or non-target siRNA control treatments for lung cancer cells. The absorbance was measured at wavelength 450 nm and 630 nm according to manufacturer’s instructions. The cell viability percentages were calculated by normalizing the survival fraction of the non-target siRNA group. Three independent experiments were performed.

### Trans-well migration and invasion assay

Migration and invasion abilities of tumor cells treated with MAP3K3 or non-target siRNAs were measured using Boyden chambers (8-mm pore size; BD Biosciences). Matrigel was purchased from BD Company. Briefly, trans-well chambers were initially coated with or without matrigel (100 μl/chamber) at 37 °C for 4 h. After being treated with MAP3K3 or non-target siRNA for 48 h, the tumor cells were harvested and resuspended at a density of 1 × 10^5^/ml in medium containing 1%FBS. The cells were then seeded at a volume of 250 μl in the upper compartment while a volume of 600 μl medium containing 10% FBS was added in the lower compartment. After incubation in a humidified atmosphere of 95% air/5% CO_2_ at 37 °C for 24 h, the non-invaded cells on the upper side of the membrane were removed with a cotton swab. The invaded cells on the bottom surface were fixed with 100% methanol and stained with hematoxylin and eosin. The stained cells were counted under an inverted microscope (5 fields per membrane). Each experiment was performed in triplicate.

### Protein and mRNA expression detection using MAPK antibody array and gene expression array

After MAP3K3 siRNA treatment for 72 h on H1299 and H838 cells, the RNA and protein were collected for Protein Array and gene expression array respectively. Non-target siRNA was used as the control group. The Human Phospho-Mitogen-activated Protein Kinase (MAPK) Antibody Array (Catalog #ARY002B) was utilized to measure reactivity to 26 different antibodies spotted on a nitrocellulose membrane according to the manufacturer’s instructions. Quantitation of Pixel densities were then determined using Image J software. Affymetrix Human Gene ST2.1 exon arrays were utilized to detect the RNA expression with mRNA isolated using Qiagen miRNA columns and the mRNA hybridized and processed by the University of Michigan microarray core.

### Statistical analysis

Data were analyzed using GraphPad Prism 6 (GraphPad software), the IBM SPSS Statistics 22 or R software. Survival curves were plotted using the Kaplan-Meier method, and survival differences were assessed by the log-rank test using the median of *MAP3K3* mRNA as a cutoff (or low vs. high score for protein). Univariate or multivariate (adjusted by age, gender, stage and differentiation) Cox proportional hazards models were calculated considering *MAP3K3* mRNA as a continuous variable. Spearman’s correlation coefficients were used for correlations between *MAP3K3* mRNA and other genes using the Shedden *et al.*, 442 ADC dataset. To identify pathway/gene expression patterns, an unsupervised hierarchical cluster analysis with uncentered average linkage was performed using Cluster v3.0 after mean-centering genes and arrays and heat maps were visualized using TreeView software^50^. The correlations between MAP3K3 protein expression on TMA and the clinical-pathological parameters were analyzed using the chi-square test. The other data such as proliferation were evaluated by unpaired Student’s t-test. A two-tailed *P* value <0.05 was considered significant. To determine potential underlying biological processes associated with *MAP3K3* correlated or regulated genes, Gene Ontology enrichment analysis was performed based on significantly correlated genes using the DAVID bioinformatics website^32^.

## Additional Information

**How to cite this article**: He, Y. *et al.* MAP3K3 expression in tumor cells and tumor-infiltrating lymphocytes is correlated with favorable patient survival in lung cancer. *Sci. Rep.* **5**, 11471; doi: 10.1038/srep11471 (2015).

## Supplementary Material

Supplementary Information
