# Supplementary material for: MAP3K3 expression in tumor cells and tumor-infiltrating lymphocytes is correlated with favorable patient survival in lung cancer
Source: Sci Rep. 2015 Jun 19;5:11471. doi: 10.1038/srep11471 (PMC4650617; doi:10.1038/srep11471)
Supplement: Supplementary Information [file srep11471-s1.pdf]

MAP3K3 expression in tumor cells and tumor-infiltrating lymphocytes is correlated with favorable patient survival in lung cancer

Yanli He[1], Lihui Wang[2], Weijun Liu[3], Jinjie Zhong[4], Shengbin Bai[4], Zhuwen Wang[5], Dafydd G. Thomas[6], Jules Lin[5], Rishindra M. Reddy[5], Nithya Ramnath[7], Philip W. Carrott, Jr. [5], William R. Lynch[5], Mark B. Orringer[5], Andrew C. Chang[5], David G. Beer[5] & Guoan Chen[5]

[1]University of Guangzhou Chinese Medicine, Guangzhou, China, [2]Guangxi Medical University, Nanning, China, [3]The First People˛s Hospital of Yunnan Province, Kunming, China, [4]Xinjiang Medical University, Xinjiang, China, [5]Section of Thoracic Surgery, Department of Surgery, [6]Department of Pathology, [7]Division of Medical Oncology, Department of Internal Medicine, University of Michigan, Ann Arbor, Michigan, 48109, United States

Correspondence and requests for materials should be addressed to D.B. (dgbeer@med.umich.edu) or G. C. (guoanche@med.umich.edu)

Supplementary Table S1-S5

Supplementary Figure S1-S7

Supplementary Table S1. MAP3K3 protein expression and clinical-pathological variables in lung adenocarcinomas (ADC)

Variables	Number	MAP3K3 Tumor		*P* value
		Score 0+1	Score 2+3	
**ADC**	88	46(52.3)	42(47.7)	
**Age (years)**				
≤65	32	16(50.0)	16(50.0)	
>65	56	30(53.6)	26(46.4)	0.75
**Gender**				
Female	44	19(43.2)	25(56.8)	
Male	44	27(61.4)	17(38.6)	0.09
**Differentiation**				
Well	26	9(34.6)	17(65.4)	
Moderate	33	18(54.5)	15(45.5)	
Poor	29	19(65.5)	10(34.5)	**0.02**
**Tumor size (T)**				
T1	39	18(46.2)	21(53.8)	
T2+T3+T4	49	29(59.2)	20(40.8)	0.31
**Lymph Node metastasis (N)**				
No	63	32 (50.8)	31(49.2)	
Yes	25	15(60)	10(40)	0.36
**Stage**				
I	54	28(51.9)	26(48.1)	
II	13	8(61.5)	5(38.5)	
III	21	11(52.4)	10(47.6)	0.97
**MAP3K3 Lymphocyte staining**				
No lymphocytes	45	31(68.9)	14(31.1)	
positive	43	16(37.2)	27(62.8)	**0.0006**

Supplementary Table S2. Cox model results for MAP3K3 mRNA in ADC

	Univariate Cox model		Multivariate Cox model*	
	**HR**	**p value**	**HR**	**p value**
Shedden data[1]	0.47 (0.19-0.75)	0.001	0.58 (0.35-0.94)	0.03
Okayama data[2]	0.30 (0.14-0.67)	0.002	0.20 (0.07-0.58)	0.003
qRT-PCR data	0.55 (0.31-0.97)	0.025	0.47 (0.25-0.89)	0.02

* age, gender, stage and differentiation were adjusted (Okayama data doesn't have differentiation information, so age, gender and stage were adjusted in this ata set).

Supplementary Table S3. MAP3K3 fusion genes

5' gene	3' gene	Tissue	Reference
MRC2	MAP3K3	Lung cancer	Dhanasekaran[3]
BCAS3	MAP3K3	Lung cancer	Seo[4]
MAO15B	MAP3K3	Brease cancer	Dan[5]
MAO15B	MAP3K3	Brease cancer	Fan[6]
MAP3K3	DDX42	Schizophrenia	Rippey[7]
TNS3	MAP3K3	mesothelioma	Panagopoulos[8]

Supplementary Table S4. Clinical characteristics of samples used in this study.

Data set	Shedden[1]	Okayama[2]	Hou[9]	Validation set
Platform	U133A	U133 plus2.0	U133 plus2.0	qRT-PCR
Sample number	442	246	156	101
Type of cancer	ADC	226 ADC,20N	65N,45ADC,27SCC,19LLC	ADC
Age average (SD)	64.4 (10.1)	64.8 (9.6)	62.9 (10.8)	67.0 (9.6)
Gender				
Female	218 (49.7%)	48 (43.2%)	48(30.8%)	53(52.5%)
Male	221	63	108	48
Stage				
Stage I	276(62.9%)	67(63.2%)	NA	59(58.4%)
Stage II	104	18	NA	16
Stage III	59	21	NA	26
Differentiation				
Well	60	NA	NA	28
Moderate	208	NA	NA	38
Poor	166(38.3%)	NA	NA	34(33.7%)
Dead (5 year)	186(42.4%)	58(52.3%)	NA	44(43.6%)
Alive	253	53	NA	57
Median survival (m)	47	31.1	NA	28.8

Abbreviation: ADC, adenocarcinoma; SCC, squamous cell cancer; LCC, large cell cancer; N, normal lung tissue.

Supplementary Table 5. Two lung tissues RNA-seq data sets

	Seo[4]	TCGA[10]
Normal	77	73
ADC	85	309
SCC	0	212

References

1. **Shedden, K., *et al.* Gene expression-based survival prediction in lung adenocarcinoma: a multi-site, blinded validation study. *Nat Med* 14, 822-827 (2008).**
2. **Okayama, H., *et al.* Identification of genes upregulated in ALK-positive and EGFR/KRAS/ALK-negative lung adenocarcinomas. *Cancer Res* 72, 100-111 (2012).**
3. **Dhanasekaran, S.M., *et al.* Transcriptome meta-analysis of lung cancer reveals recurrent aberrations in NRG1 and Hippo pathway genes. *Nat Commun* 5, 5893,doi: 10.1038 (2014).**
4. **Seo, J.S., *et al.* The transcriptional landscape and mutational profile of lung adenocarcinoma. *Genome Res* 22, 2109-2119 (2012).**
5. **Robinson, D.R., *et al.* Functionally recurrent rearrangements of the MAST kinase and Notch gene families in breast cancer. *Nat Med* 17, 1646-1651 (2011).**
6. **Fan, Y., *et al.* Amplification and over-expression of MAP3K3 gene in human breast cancer promotes formation and survival of breast cancer cells. *J Pathol* 232, 75-86 (2014).**
7. **Rippey, C., *et al.* Formation of chimeric genes by copy-number variation as a mutational mechanism in schizophrenia. *Am J Hum Genet* 93, 697-710 (2013).**
8. **Panagopoulos, I., Gorunova, L., Davidson, B. & Heim, S. Novel TNS3-MAP3K3 and ZFPM2-ELF5 fusion genes identified by RNA sequencing in multicystic mesothelioma with t(7;17)(p12;q23) and t(8;11)(q23;p13). *Cancer Lett* 357,502-509(2015).**
9. **Hou, J., *et al.* Gene expression-based classification of non-small cell lung carcinomas and survival prediction. *PLoS One* 5, e10312, doi: 10.1371 (2010).**
10. **Comprehensive molecular profiling of lung adenocarcinoma. *Nature* 511, 543-550 (2014).**

Supplementary Figure S1

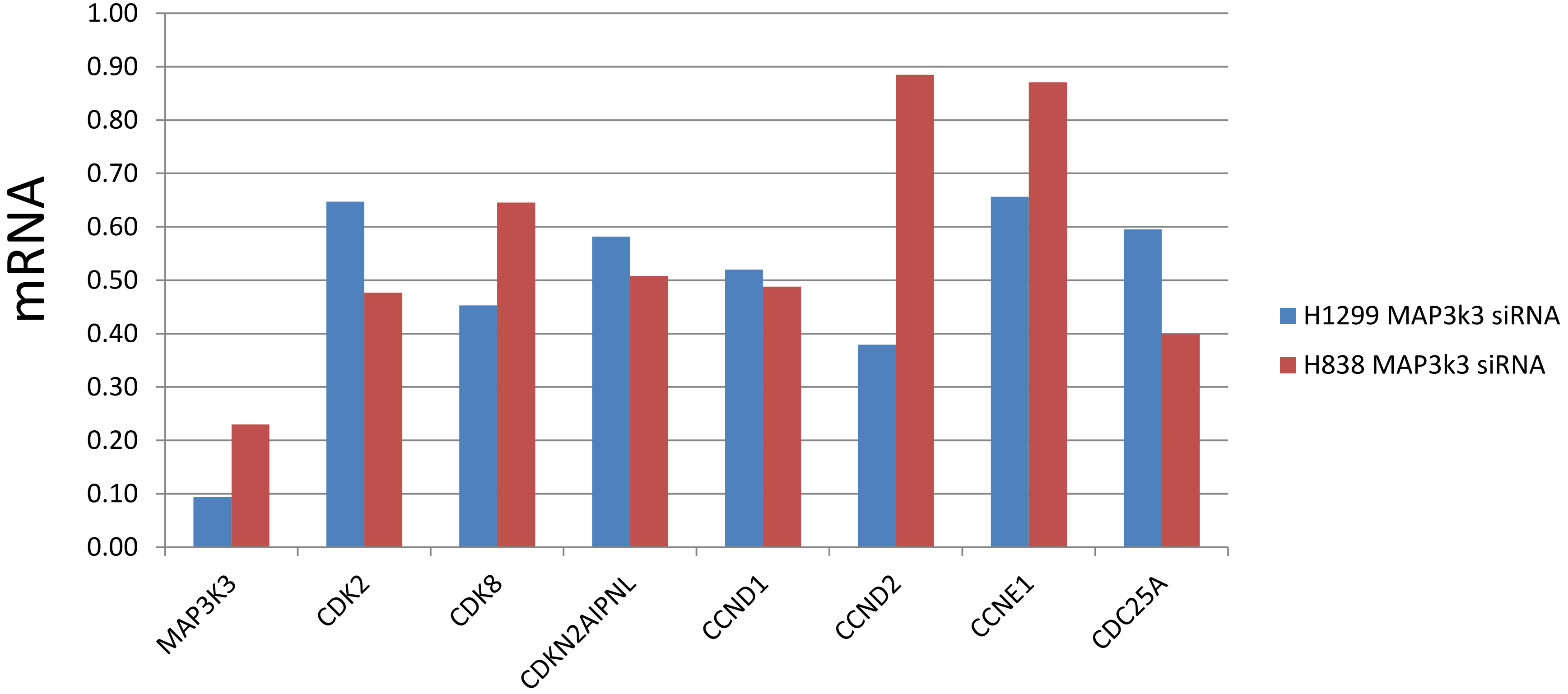

Supplementary Figure S1. Cell Cycle related genes are decreased after MAP3K3 siRNA knockdown using Affymetrix Exon ST2.1 array. Further validation by Western blot is warrant.

Supplementary Figure S2

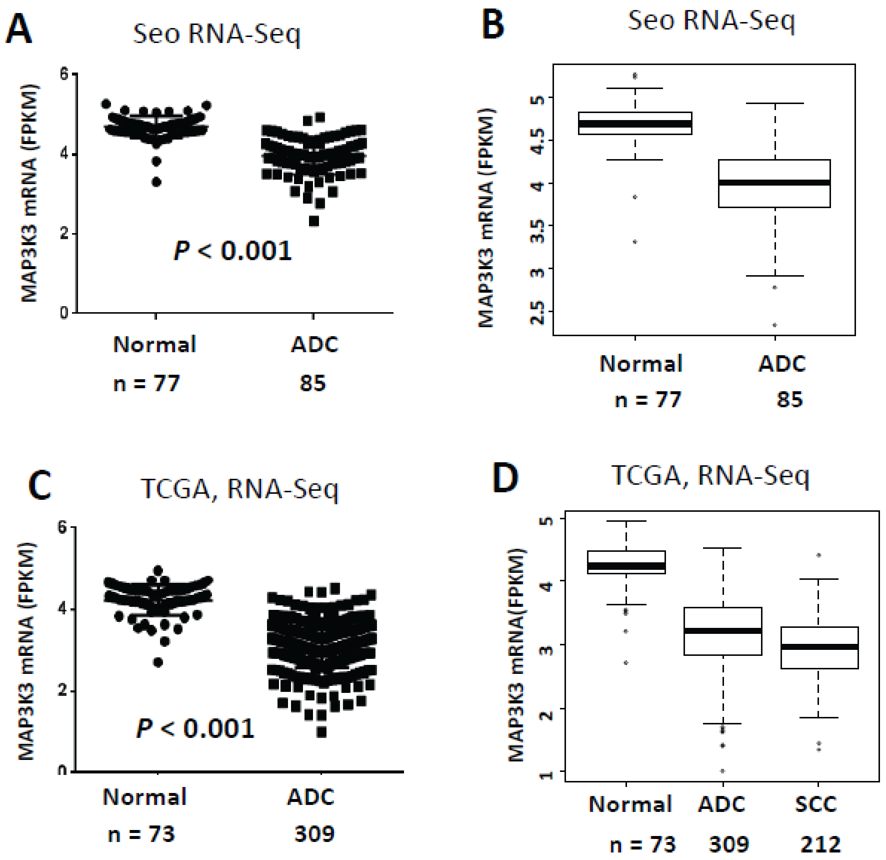

Supplementary Figure S2. *MAP3K3* mRNA was higher in normal lung tissues compared to lung cancer in two RNA-Seq data sets.

Supplementary Figure S3

Oncomine Disease Summary for MAP3K3

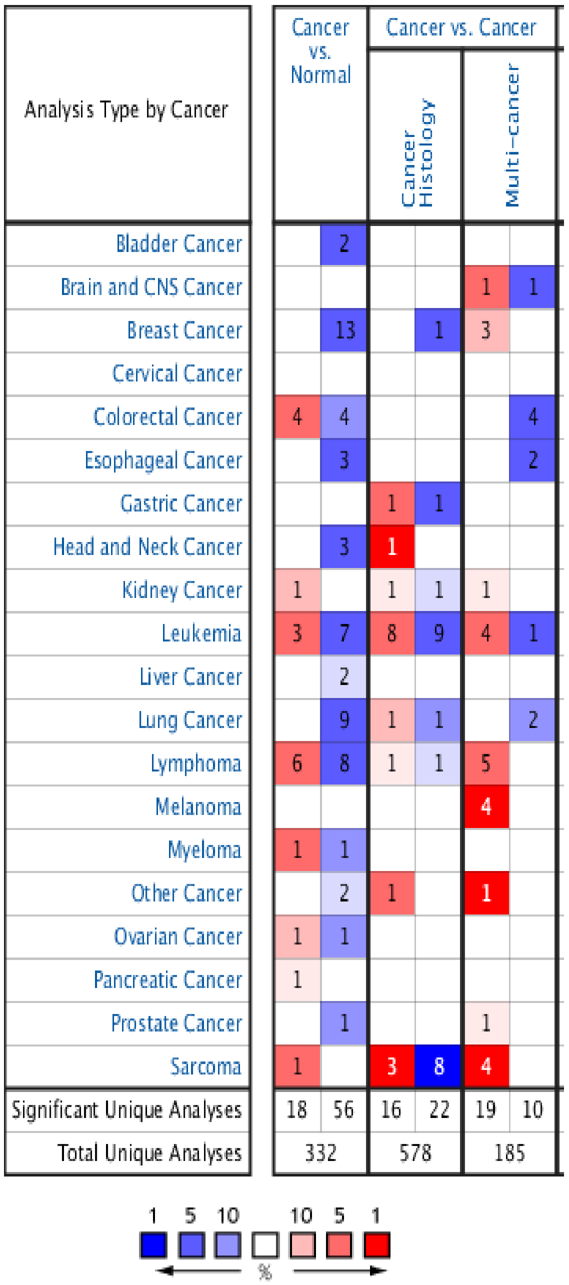

Supplementary Figure S3. From Oncomine data base: *MAP3K3* mRNAs were decreased in many kinds of cancers (vs. normal) including bladder, breast, esophagus, Head & Neck,liver, lung and prostate cancers. Whereas, colorectal cancer, leukemia, lymphoma, myeloma and ovarian cancer were not consistent from different studies. Red= higher in tumor (vs. Normal), Blue= lower in tumor. Number indicated number of data set or comparison between tumor vs. normal.

Supplementary Figure S4

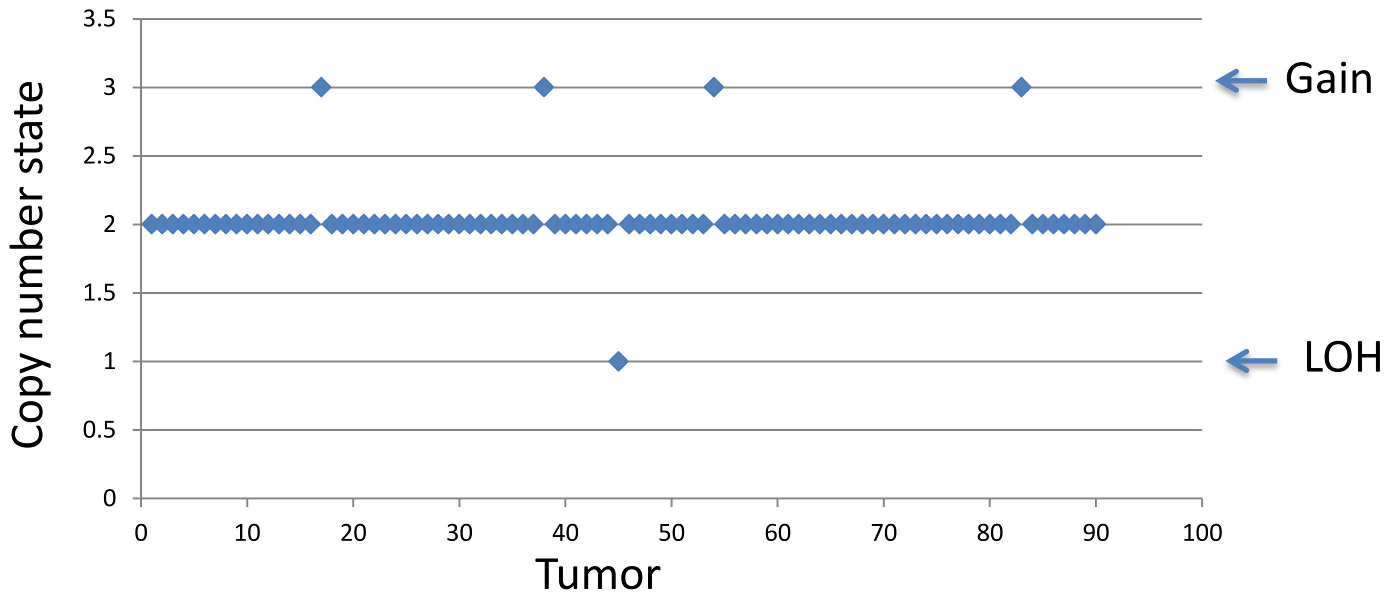

Supplementary Figure S4.MAP3k3 (located at chr17:61609801-61773670) DNA copy number change in 90 lung adenocarcinomas using SNP6.0, analyzed using Affymetrix Genotyping Console (GTC 4.1). 4 single copy gain and 1 heterozygous deletion were found.

State 0 = CN of 0; homozygous deletion
State 1 = CN of 1; heterozygous deletion
State 2 = CN of 2; normal diploid
State 3 = CN of 3; single copy gain
State 4 = CN 4; amplification

Supplementary Figure S5

Sample ID	AA Mutation	CDS Mutation	Pubmed Id
1423679	p.R597W	c.1789C>T	18948947
1423766	p.A510fs*25	c.1528delG	18948947
1423831	p.R434L	c.1301G>T	18948947
1863687	p.R299Q	c.896G>A	22975805
1863732	p.G272D	c.815G>A	22975805
2015226	p.P216S	c.646C>T	23733853
1780065	p.R363L	c.1088G>T	-
1780081	p.P160P	c.480C>T	-
2024537	p.R597L	c.1790G>T	23799614
1782659	p.R363L	c.1088G>T	-
1759434	p.?	c.779-2A>T	22941189
1870263	p.R608H	c.1823G>A	23033341
1759264	p.?	c.779-2A>T	22941188
1765262	p.A338P	c.1012G>C	22980975
1914116	p.L94V	c.280C>G	-
1914083	p.T462K	c.1385C>A	-

Supplementary Figure S5. MAP3K3 gene mutations were detected in 16 out of 1218 (1.3%) lung cancers. http://cancer.sanger.ac.uk/cosmic

Supplementary Figure S6

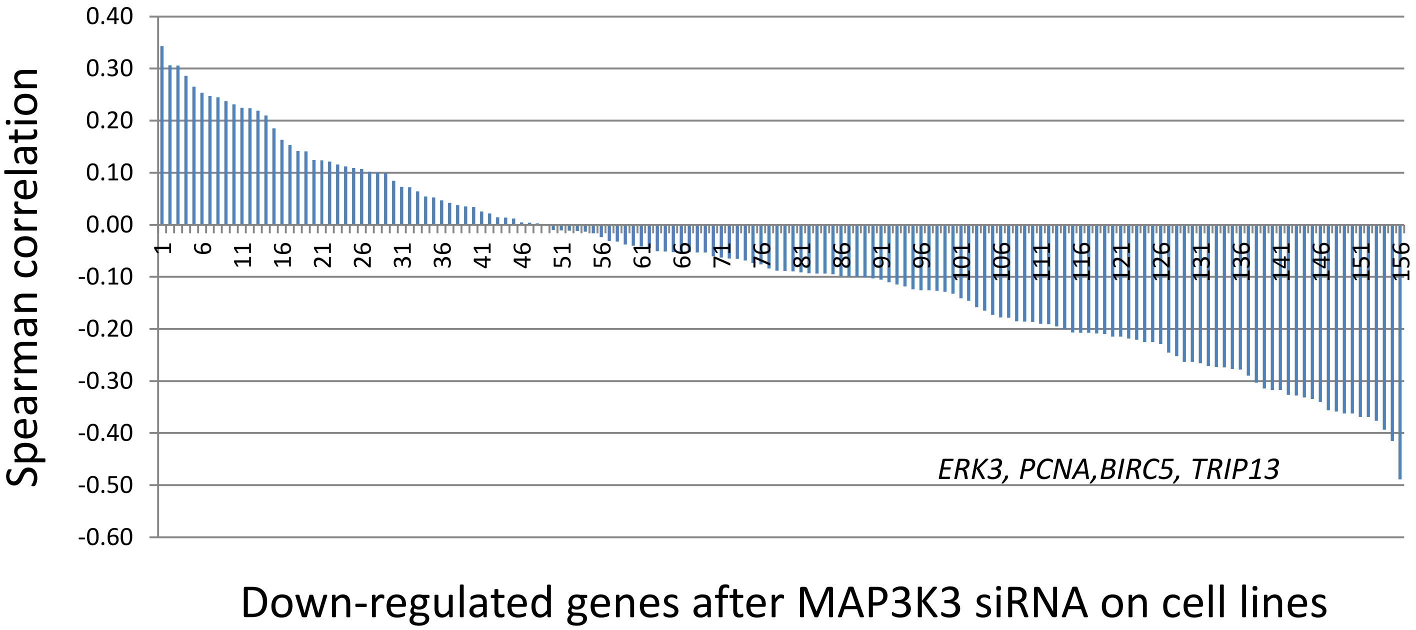

Supplementary Figure S6. Most (107/156) down regulated genes (0.6 fold after MAP3K3 siRNA treatment) on H1299 and H838 cell lines (*in vitro*) are lost or became negatively correlated to MAP3K3 on 442 ADC (*in vivo*) may be due to the tumor environment changes such as *ERK3, PCNA, BIRC5* and *TRIP13*, etc.

Supplementary Figure S7

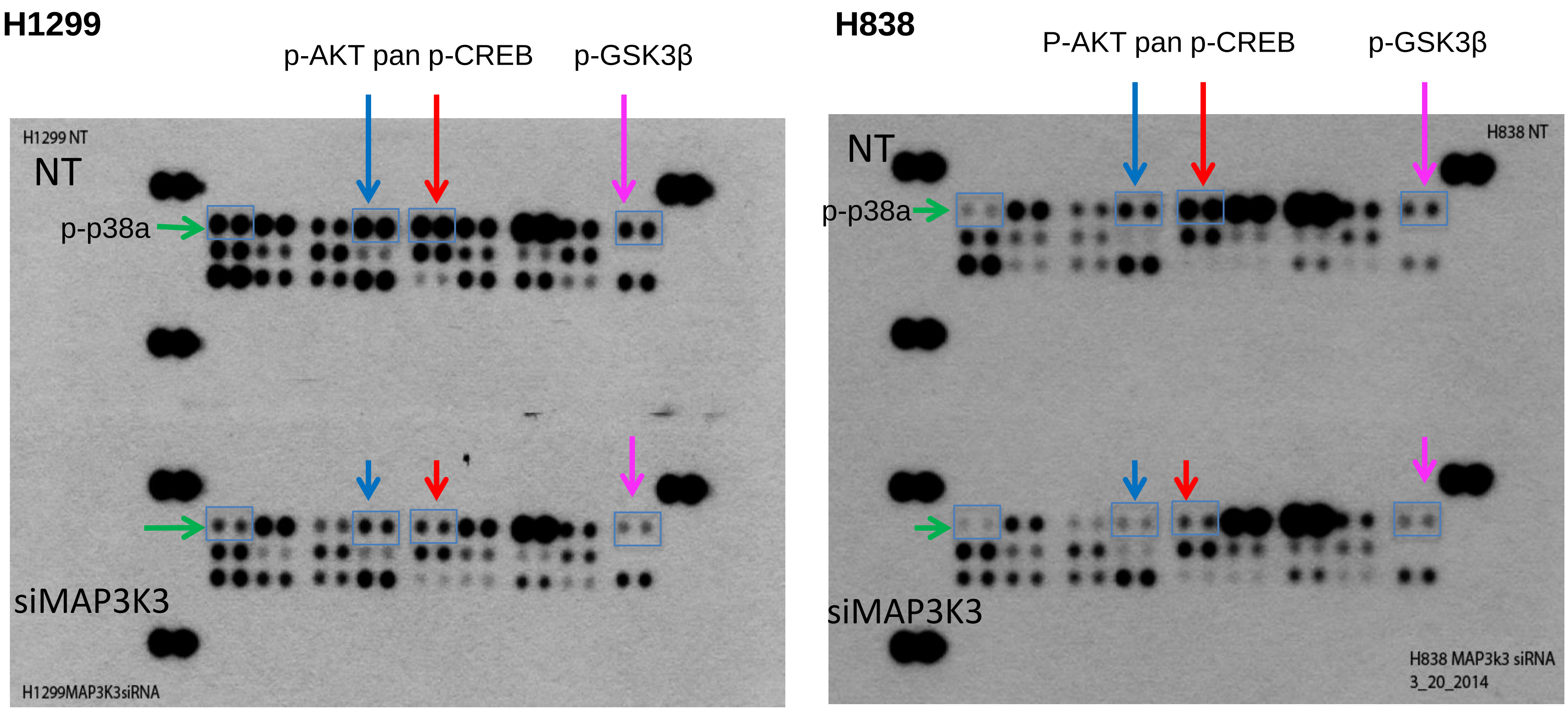

Supplementary Figure S7. Original image for main Figure 2A.
Four phospho-proteins (p-AKTpan, p-GSK3β, p-P38α and p-CREB) were down-regulated after MAP3K3 siRNA knockdown (siMAP3K3) at 72 h in both the H1299 and H838 cell lines as compared to non-target siRNA control (NT) as determined using the Human MAPK antibody array
